# Comparison of end-diastolic versus end-systolic cardiac-computed tomography reconstruction interval in patient’s prior to pulmonary vein isolation

**DOI:** 10.1186/2193-1801-3-218

**Published:** 2014-05-01

**Authors:** Wieland Staab, Sabrina Goth, Christian Sohns, Jan Martin Sohns, Michael Steinmetz, Christina Unterberg Buchwald, Andreas Schuster, Johannes Tammo Kowallick, Martin Fasshauer, Joachim Lotz

**Affiliations:** Department of Diagnostic and Interventional Radiology, Georg-August-University, Robert-Koch-Str. 40, 37075 Goettingen, Germany; Department of Cardiology and Pneumology, Georg-August-University, Goettingen, Germany; DZHK, Göttingen, Germany

**Keywords:** PVI, Thrombus, LAA, Volumes

## Abstract

**Purpose:**

Aim of the study was to investigate diagnostic accuracy of cardiac computed tomography angiography (CCTA) between left ventricular end-systolic (LVES) and left ventricular end-diastolic (LVED) cardiac phase for thrombus detection in patient’s prior to pulmonary vein isolation (PVI).

**Materials and methods:**

182 consecutive Patients with drug refractory AF scheduled for PVI (62.6% male, mean age 64.1 ± 10.2 years) underwent routine pre-procedural evaluation including transesophageal echocardiography (TEE) and CCTA for evaluation of left atrial (LA)/left atrial appendage (LAA) anatomy and thrombus formation. Qualitative and quantitative analysis (using aorta ascendens (AA)/LAA ratio) was performed. Measurements of the LA/LAA in LVES and LVED cardiac phase were obtained.

**Results:**

End-systolic volumes (LA/LAA) measured in 30 patients without filling defects as control group and all 14 with filling defects of 182 patients were significantly larger (p < 0.01) than in end-diastolic phase. Qualitative analysis was inferior to quantitative analysis using LA/LAA ratio (<0.5; accuracy: 100%, 88%,100%, 99% vs 100%). 5 out of 182 patients (2.7%) showed thrombus formation of the LAA in CCTA confirmed by TEE and quantitative analysis. Intra/-interobserver variability was lower in end-systolic vs end-diastolic reconstruction interval.

**Conclusion:**

For evaluating CCTA datasets in patients prior PVI, the LVES reconstruction interval is recommended due to significantly larger LA/LAA volumes and lower intra/- interobserver variability’s.

## Introduction

Atrial fibrillation (AF) is the most common sustained cardiac arrhythmia with a significant increase of morbidity and mortality due to its associated risk of thrombembolism. Pulmonary vein isolation (PVI) by percutaneous radiofrequency ablation has emerged as a therapeutic option. Actually it is recommended for symptomatic and drug refractory patients (Romero et al. 2013). In order to prevent thrombembolic events, it is mandatory to exclude left atrial (LA) and LA appendage (LAA) thrombus prior to PVI (Dorenkamp et al. 2011; Scherr et al. 2009; Hur et al. 2009). Transesophageal echocardiography (TEE) is considered the gold standard modality in detecting left atrial (LA)/LA appendage (LA/LAA) thrombi (Dorenkamp et al. 2011; Scherr et al. 2009). However, this is a semi-invasive procedure with rare but potential life-threatening complications (Scherr et al. 2009). Cardiac computed tomography angiography (CCTA) has been proposed as an alternative method (Romero et al. 2013; Dorenkamp et al. 2011; Scherr et al. 2009). Prior to PVI, CCTA and TEE are used to provide exact anatomical details of the size of the left atrium as well as the number and position of the pulmonary veins entering the left atrium and to exclude LA/LAA thrombus (Gage et al. 2004). Reported limitations of CCTA are the high false positive rate of thrombus formations and therefore low specificity, unacceptable interobserver variability’s (Aljaroudi et al. 2013).

Prospective electrocardiography (ECG) triggering became available with newer scanner techniques, allowing data acquisition during a pre-defined, narrow portion of the R–R interval resulting in a reduction in radiation dose (Steigner et al. 2009). Here, the mid-to-end-diastole at 70 - 75% of the R-R interval is mostly used for prospective-ECG- triggering, since coronary artery motion is small in that phase (Menke et al. 2013). In the traditional retrospective ECG-gated-CCTA method, the full cardiac cycle is imaged (0-100%), and the heart is retrospectively reconstructed at the required cardiac phases (Dewey 2011). In this context, retrospective gating is chosen because temporal as well as dynamic information is obtained and CCTA images can be reconstructed at other cardiac phases (0-100%), if the standard phase was not diagnostic or interpretable (Steigner et al. 2009; Menke et al. 2013). Therefore, we can choose appropriate phases for minimum and maximum LA/LAA diameters and volumes at end-diastole and end-systole for post-processing and evaluating CCTA data. Aim of the study was to investigate diagnostic performance of thrombus detection in CCTA between cardiac cycles, intra/- interobserver variability’s of thrombus detection as well as comparing acquired volumetric and diametric datasets.

## Materials and methods

### Patients

182 consecutive prospectively evaluated patients with AF scheduled for PVI (62.6% male, mean age 64.1 ± 10.2 years, Table 1) underwent routine diagnostic work up prior to PVI including TEE and CCTA (from January 2010 until May 2013). In all cases CCTA and TEE were performed within 1–3 days prior to PVI. The analysis was approved by the local ethic committee (University Medical Center Göttingen). The CHADS2 scoring system (Romero et al. 2013; Scherr et al. 2009; Camm et al. 2012) was used for risk stratification of thromboembolic events. The scoring system assigns one point for the presence of age >75, heart failure, diabetes mellitus and hypertension and two points for prior transitory ischemic attack (TIA) or stroke. Further risk factors of LA/LAA thrombus such as chronic kidney disease, valvular disease, cardiomyopathy and LA size were evaluated and documented (Camm et al. 2012), Table 2.

**Table 1:** **Patient demographic characteristics**

**Male**	114 (62.6%)
**Female**	68 (37.4%)
**Age, mean**	64.1 ± 10.2
**Age ≥ 75 years**	15 (8.2%)
**Mean ejection fraction**	54.2 ± 6.1
**Paroxysmal atrial fibrillation**	119 (65.4%)
**Persistent atrial fibrillation**	63 (34.6%)
**Cardiomyopathy (dilated)**	9 (4.9%)
**Mitral/aortic valve disease**	29 (15.9%)
**Serum creatinine; mg/dl**	0.94 ± 0.27
**Elevated serum creatinine**	27 (14.8%)
**CHADS 2 score:**	
**CHADS2 = 0**	41 (22.5%)
**CHADS2 = 1**	92 (50.5%)
**CHADS2 = 2**	36 (19.8%)
**CHADS2 ≥ 3**	13 (7.1%)

**Table 2:** **Clinical characteristics of patients with and without LA/LAA thrombus**

	No LA/LAA thrombus	LA/LAA thrombus	p-value
	N = 177 (97.3%)	N = 5 (2.7%)	
Male	110 (62.1%)	4 (80%)	n.s.
Female	67 (37.9%)	1 (20%)	n.s.
Age, mean	64.0 ± 8.8	68.8 ± 12.9	0.01
Mean ejection fraction	54.3 ± 6.0	48.6 ± 7.7	n.s.
Paroxysmal atrial fibrillation	116 (65.5%)	3 (60%)	n.s.
Persistent atrial fibrillation	61 (34.5%)	2 (40%)	n.s.
Cardiomyopathy (dilated)	9 (5.1%)	0 (0%)	n.s.
Mitral/aortic valve disease	27 (15.3%)	2 (40%)	n.s.
Serum creatinine; mg/dl	0.93 ± 0.27	1.12 ± 0.20	n.s.
Elevated serum creatinine	25 (14.1%)	2 (40%)	n.s.
*CHADS 2*			
Congestive heart failure	33 (18.6%)	1 (20%)	n.s.
Hypertension	111 (62.7%)	4 (80%)	n.s.
Diabetes mellitus	30 (16.9%)	4 (80%)	0.0004
Prior stroke or TIA	7 (4.0%)	0 (0%)	n.s.
Age ≥ 75 years	12 (6.8%)	3 (60%)	<0.0001
CHADS2 = 0	41 (23.2%)	0 (0%)	n.s.
CHADS2 = 1	92 (52.0%)	0 (0%)	0.009
CHADS2 = 2	33 (18.6%)	3 (60%)	0.02
CHADS2 ≥ 3	11 (6.2%)	2 (40%)	0.004

CHADS2: congestive heart failure, hypertension, age > 75 years, diabetes and stroke (doubled).

### Computed tomography

CCTA was performed with a 64-slice MDCT scanner (VCT LightSpeed, GE Healthcare, Milwaukee, WI, USA), slice collimation 64 × 0.625 mm; rotation time 600 msec; tube voltage 100–120 kV; adaptive dose regime (auto mAs, 280–380). Depending on the scan range and the patient’s body weight the calculated mean radiation dose was 6.1 ± 2.6 mSv (dose-length product range, 137 to 537 mGy * cm multiplied with the conversion coefficient for the chest of k = 0.017 mSv mGy^-1^ cm ^-1^). Retrospective ECG gated half scan algorithm was used to reconstruct the data into contiguous axial images with a slice thickness of 0.625 mm. For this study, images were evaluated at end- diastolic as well as end-systolic cardiac cycle (39 ± 4% and 77 ± 5% of the RR-interval) on a dedicated workstation (Aquarius 3D Workstation, TeraRecon, San Mateo, CA, USA). Beta adrenergic blocking agents were administered prior the scan procedure if the heart rate was > 70 bpm (79% of the investigated patients) (mean heart rate 61 ± 8 bpm). Contrast media injection protocol (split-bolus) was as followed: injection of 30 ml at 2 ml/sec of iodinated intravenous contrast agent (Imeron 350, Bracco, Konstanz, Germany); a 20 sec break followed by 70 ml at 4 ml/sec of the same contrast and a 40 ml saline chaser at 4 ml/sec in a single spiral scan technique within a single breath-hold covering an area from the aortic arch to below the diaphragm. Semi-automatic bolus chasing was used to detect the second contrast bolus in the ascending aorta. CCTA images were analyzed separately at 39 ± 4 and 77 ± 5% RR-interval (quantitative and qualitative analysis at end-diastolic as well as end-systolic cardiac cycle independently by both readers) blinded to TEE results by two experienced independent readers. Complete CCTA Datasets were evaluated by both readers independently at two different time periods. Qualitative, visual assessment of the LA/LAA was done using on out of three categories: no-thrombus, contrast filling defect or definite thrombus formation. A filling defect was defined as an intracavitary low attenuating round or oval lesion representing incomplete mixing of contrast agent and blood, whereas thrombus formation was defined as round-or oval shaped low attenuation (LAA/AA ratio < 0.5) area (Romero et al. 2013; Hur et al. 2009; Hur et al. 2013). Quantitative measurement of relative contrast enhancement of the LA and LAA to the ascending aorta was done in all patients. Here, a 1 cm^2^ region of interest (ROI) was placed inside the filling defect in the LAA seen on CCTA images and the AA to generate the LAA/AA-ratio. In case no filling defect was observed in CCTA images, a 1 cm^2^ ROI was as well placed in the LAA and the AA for calculating LAA attenuation to AAo attenuation values. A cut-off value of LAA/AA ratio < 0.5 was used to differentiate between thrombus and filling defect on CCTA.

### Echocardiography

Transesophageal echocardiography (TEE) as well as transthoracic echocardioagraphy (TTE) was performed in all patients. TEE was obtained using a GE Vivid E9 ultrasound system (General Electric Ultrasound, Horten, Norway) with a 5.0 MHz multiplane probe acquiring continuous cine loops of LA/LAA in 0° -180°. TTE was used to determine left ventricular ejection fraction and LA size. Images were acquired according to the methods described by the American Society of Echocardiography from parasternal long and short axis, long axis, apical four chamber and two chamber views (Camm et al. 2012). Highly experienced (at least 4 years experience) cardiologists performed and interpreted all TEEs blinded to patient’s history and results from other procedures such as CCTA. Thrombus was defined as a distinct intracavitary echo-lucent or echo-dense mass in comparison to dense non-clearing spontaneous echo contrast (SEC), defined as a slow swirling smoke like echo -density (Scherr et al. 2009; Hur et al. 2009; Hur et al. 2013). The teams of TEE/TTE and CCTA were blinded to the results of the other modality during the assessment of LA/LAA thrombus formation.

### Definitions and measurements of LA/LAA

**Figure 1:**
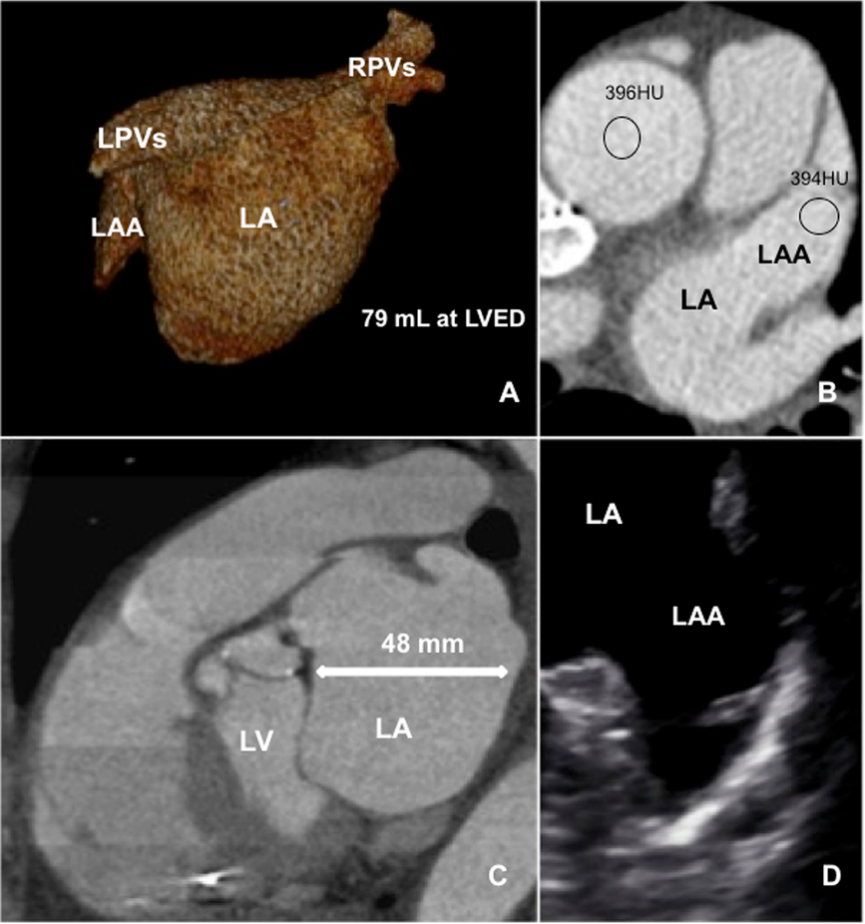
**3 – Dimensional VR (A) with measured LA volume using the Simpsons method at LVED from a 71 year old female patient with paroxysmal AF prior PVI; CCTA (B) demonstrates trabeculated fully opacified LAA with LAA/AA ratio from 0.99; CCTA (C) reconstructed in oblique sagittal view showing LA at LVED on a dedicated workstation using TeraRecon ©; Corresponding TEE (D) shows trabecularisation of the LAA without SEC or thrombus.**

Reformats were done on a dedicated workstation (Aquarius 3D Workstation, TeraRecon®, San Mateo, CA, USA) (Figure 1). Minimal left atrial volume (LAV) was defined at left ventricular (LV) end-diastole (ED) when the LV was largest and LA smallest whereas maximum LAV was defined at LV end-systole (ES) directly before mitral valve opening when LAV was largest. The left atrial appendage (LAA) was evaluated separately. LAV was quantified according to a modified Simpson’s method (Stojanovska et al. 2011). For quantifying LA diameters in sagittal, coronal and axial direction, reformatted images were obtained at the level of the aortic valve parallel to the LV outflow tract in LVES for maximum LA diameters and in LVED for minimum LA diameters. LAA was measured according to this method for minimal (LVED) and maximum (LVES) diameters and volumes. As a statistical significant homogenous group, 14 patients with detected thrombus or filling defect of LAA on CCTA (Table 2) were assessed and 30 patients without filling defects on CCTA 15 male (50%), 15 with persistent AF (50%), mean age 62.3 ± 5.6 were evaluated as control group. Intra/-intraobserver variability’s for data reproducibility was independently obtained.

### Statistical analysis

Biostatistics were planned and performed by the local Department of Medical Statistics. Descriptive statistical analyses, variables were expressed as the means ± standard deviations and/or percentages. Intra/-interobserver agreement for LA/LAA thrombus was calculated using Cohen’s kappa (κ) statistics (kappa values: poor < 0.20, fair 0.21-0.40, moderate 0.41 – 0.60, good 0.61-0.80, excellent 0.81-1.00). Student’s t-tests and Fisher’s exact tests were used to compare population averages and statistical significance of categorical population differences or Chi-squares test for eventually categorical variables for independence between groups. Sensitivity, specificity, negative (NPV) and positive predictive values (PPV) were assessed assuming TEE as reference standard for thrombus detection by using chi-squared test. For all obtained data, p < 0.05 was considered to be statistical significant. Statistical analysis was performed using SAS/STAT software (version 9.3, SAS Institute).

## Results

### Patient characteristics

Patient characteristics of all 182 consecutive patients are summarized in Table 1. Image quality of all 182 consecutive CCTA examinations was regarded to be diagnostic. Left atrial and left atrial appendage (LA/LAA) evaluation was feasible in all cases and no adverse events were reported during TEE and CCTA examination. CCTA and TEE examinations were performed within 3 days (2 days ± 1). CCTA showed 14 out of 182 patients with filling defects in the LAA. Using LAA/AAo ratio of 0.5, five patients showed definite thrombus formation, confirmed by TEE. These patients were excluded from ablation procedure. Paroxysmal AF was present in 65.4% of patients whereas 34.6% showed persistent AF. 78% of patients were treated with phenprocumon at time of CCTA and TEE exam, 17% used aspirin for anticoagulation. At time of CCTA, AF was present in 61 patients (33.5%).

### Thrombus formation in CCTA versus TEE and related characteristics

**Figure 2:**
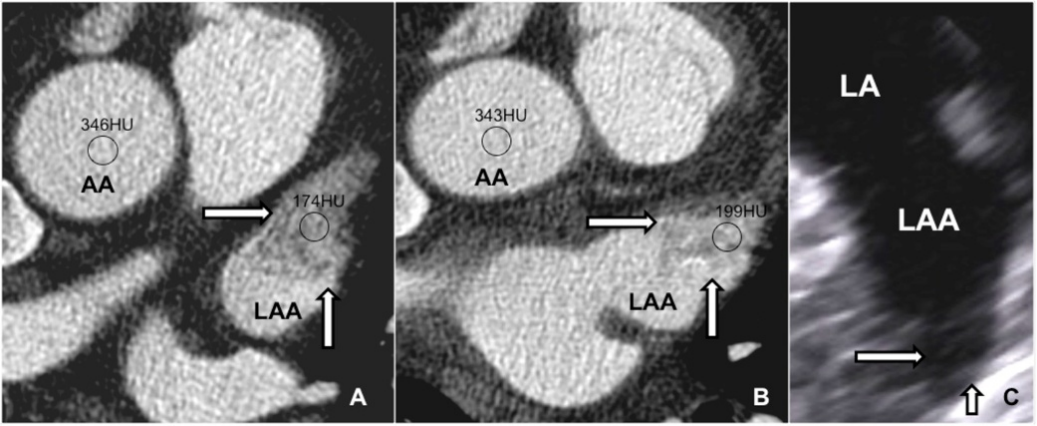
**CCTA with filling defect (arrows) at LVED (A) and LVES (B); LAA is visually and quantitatively better opacified at LVES (A) (LAA/AA ratio 0.51 (A) and 0.59 (B)) indicating SEC, excluding thrombus, confirmed by TEE (C) (arrows) in a 63 year old male patient prior PVI with persistent AF.**

**Figure 3:**
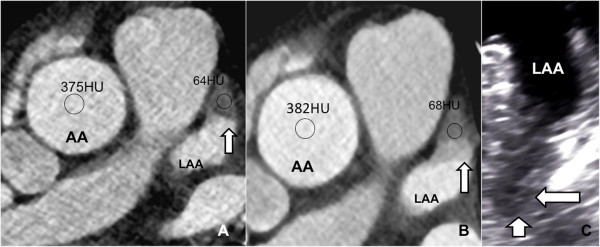
**CCTA with depicted filling defect (arrows) in the LAA at LVED (A) and LVES (B) showing thrombus formation (LAA/AA ratio 0.17 (A) vs 0.18 (B)) in a 64 year old male patient with persistent AF confirmed by TEE (C).** Additional visible SEC on top of thrombus with higher density at LVES **(B)** and TEE.

In 5 cases a definitive thrombus formation using quantitative analysis (cut-off value of LA/LAA coefficient < 0.5) was seen in the LAA confirmed by TEE (Table 2). 9 patients (3 with paroxysmal AF) showed filling defects without definitive thrombus formation using quantitative analysis (cut-off value of LA/LAA coefficient > 0.5, Figure 2) judged as thrombotic precursors in the state of circulatory stasis confirmed by TEE (all patients showed slow-flow/SEC of the LAA). TEE identified 2 additional patients with SEC in the LA or LAA that were missed by CCTA. Patients with detected LAA thrombus (5 out of 182, 2.7%, Figure 3) showed absolutely a lower mean ejection fraction (54.3 ± 6.0% vs 48.6 ± 7.7%), a higher serum creatinine (mg/dl) (0.93 ± 0.27 vs 1.12 ± 0.2) and relatively more patients with mitral or aortic valve disease (40% vs 15.3%). In concordance to the CHADS2 score, diabetes mellitus (16.9% vs 80%, p value 0.0004) and age ≥ 75 (6.8% vs 60%, p value <0.0001) were significantly more prevalent in patients with LAA thrombus. Significantly higher CHADS2 scores were present in patients with thrombus (CHADS 2 = 2; 18.6% vs 60%, p value 0.02 and CHADS2 ≥ 3; 6.2% vs 40%, p value 0.004). LA/LAA thrombus was not detected in patients with a CHADS2 score ≤ 1 and an age ≤ 50 years.

### Measurements of LA and LAA

In 30 patients without thrombus, diameters as well as volumes (absolute and indexed) were significantly larger in the LVES at 39 ± 4% RR-interval (Table 3) (p < 0.01). In 14 patients with filling defect on CCTA, diameters and volumes as well as indexed values were significantly larger in the LVES at 39 ± 4% RR-interval (p < 0.01) than in LVED. Patients with thrombus showed larger diameters and volumes as well as indexed values than patients without (p < 0.01). Measurements were done blinded to patient’s data 2 times within 6 weeks.

**Table 3:** **Measurements including diameters, volumes and indexed values of diameters and volumes showing significant differences in volumes and diameters between LVED und LVES**

Measurement	30 Patients without filling defect at LVED	30 Patients without filling defect at LVES	p-value	14 Patients with filling defect at LVED	14 Patients with filling defect at LVES	p-value
Diameter- axial (mm)	75.90+/- 7.95	87.10+/- 8.81	< 0.01	80.25+/- 7.74	88.85+/- 8.45	< 0.01
Diameter – sagittal (mm)	38.25+/- 6.6	53.10+/- 7.23	< 0 .01	46.70+/- 5.45	54.50+/- 6,77	< 0.01
Diameter – coronar (mm)	74.05+/- 9.3	79.90+/- 5,16	< 0.01	78.70+/- 9.32	85.50+/- 6.72	< 0.01
Volume- LA (mL)	169.50+/- 31.62	176.51+/- 34.57	< 0.01	187.00+/- 32.87	192.5+/- 33.59	0.026
Volume- LAA (mL)	6.673.45	9.39+/- 4.87	< 0.01	8.71+/- 2.24	10.72+/- 3.55	0.03
Diameter- axial/BSA (mm/m^2^)	37.64+/- 7.23	41.78+/- 6.56	< 0.01	38,83+/- 7.99	42.56+/- 6.43	< 0.01
Diameter – sagittal/BSA (mm/m^2^)	19.27+/- 4.08	26.33+/- 6.20	< 0.01	22.82+/- 5.79	26.18+/- 7.58	< 0.01
Diameter – coronar/BSA (mm/m^2^)	35.87+/- 6.06	40.16+/- 7.53	< 0.01	37.69+/- 8.01	40.941+/- 8.99	< 0.01
Volume- LA/BSA (mL/m^2^)	82.00+/- 21.68	85,80+/- 21.82	0.04	90.66+/- 23.58	93.65+/- 23.66	0.02
Volume- LAA/BSA (mL/m^2^)	3.2+/- 1.82	4.41+/- 2.23	0.035	4.14+/- 3.21	5.18+/- 2.87	0.25

### Intra-/Interobserver variability’s

Assuming TEE as standard of reference for detection of thrombus or SEC, qualitative visual CCTA analysis at 77 ± 5% of RR-interval resulted in a sensitivity of 94%, specificity of 84%, a negative predictive value (NPV) of 92% and a positive predictive value (PPV) of 87% whereas visual evaluation at 39 ± 4% RR-interval showed superior results of 100% sensitivity, 94% specificity, 100% NPV and 93% PPV for both readers in consensus. Applying a value of < 0.5 (LAA/AA ratio) relative contrast enhancement in CCTA as a threshold of thrombus-formation, the overall sensitivity, specificity, NPV and PPV were 100% independently for both readers at both dates at 39 ± 4 and 77 ± 5% RR-Interval. Intra/-interobserver variability’s were lower at absolute values at LVES in both readers (39 ± 4% RR-interval; κ = 0.942 vs 0.901) vs LVED (77 ± 5% RR-interval; κ = 0.891 vs 0.860).

## Discussion

### Main finding

Quantitative analysis (threshold of < 0.5 when using LAA/AA ratio) of CCTA filling defects is superior to qualitative visual analysis in detecting or ruling out LA/LAA thrombus. For evaluating CCTA datasets in patients prior to PVI, the LVES (at 39 ± 4% RR-interval) reconstruction interval is recommended due to significantly larger LA/LAA diameters/volumes (p < 0.01) and lower intra/- interobserver variability’s (κ = 0.942 vs κ = 0.891) in absolute values.

### Thrombus formation

To minimize the risk of periprocedural thromboembolic events during or after catheter ablation in the LA, it is necessary to rule out LA/LAA thrombus formation prior to PVI. TEE is an effective, reproducible method for excluding intracavitary thrombi and actually remains the gold-standard to assess LA/LAA thrombus formation but sedation due to the invasiveness of the modality (Camm et al. 2012). Various study protocols for detection of LA/LAA thrombus have been described and discussed in the past few years (Dorenkamp et al. 2011; Hur et al. 2009; Aljaroudi et al. 2013; Hur et al. 2013). Commonly iodinated contrast agent is used in a single-bolus injection with monophasic or biphasic (early/late phase imaging) CT protocols (Dorenkamp et al. 2011; Hur et al. 2009; Aljaroudi et al. 2013). A major limitation of CCTA is incomplete contrast filling of the LAA, representing the most frequent cause of false positive filling defects, leading to a low PPV up to 30% (Hur et al. 2009; Aljaroudi et al. 2013). Wazni et al. (Wazni et al. 2007) reported no increase of peri-procedural stroke if SEC was identified in TEE. Additionally, even appropriate anticoagulation did not have an influence on presence of SEC (Wazni et al. 2007). In the present study, 9 out of 11 patients with SEC in TEE had non-thrombotic filling defects in CCTA in the LAA with a LAA/AA ratio >0.5. The frequency of 2.7% intracardiac thrombus formations prior PVI is in line with previous studies (Scherr et al. 2009). Here, pre-saturation of the blood pool using a split-bolus injection single-scan protocol seems to decrease non-thrombotic filling defects by increasing contrast filling of the LAA and thus improving PPV. CCTA is not only able to evaluate LA/LAA region, pulmonary veins but can as well identify coronary artery placque and calcification with high sensitivity and specificity for diagnosing coronary artery disease (CAD) in patients with AF prior to PVI (Sohns et al. 2012).

### Measurements of LA and LAA

Evaluation of LA volume and diameter is crucial prior to PVI to adequately assess patients eligible for the procedure as well as after ablation procedure to observe or exclude post-procedural complications (Hur et al. 2009; Stojanovska et al. 2011). Larger LA volumes and/or diameters due to AF and LA/LAA thrombi are associated with an increased risk of stroke and other embolic events (Hur et al. 2009; Gage et al. 2004; Stojanovska et al. 2011). The American Society of Echocardiography and the European Association for Echocardiography proposed the Simpsons method or the biplane area length method for evaluation of LA volume and diameter (Calkins et al. 2012). The Simpsons method is recommended while it does not rely on geometric assumptions and LAA as well as the ostia of the pulmonary veins are excluded from analysis (Christiaens et al. 2009). Absolute as well as indexed values of mean LA volume and diameter for all ages were in line with previous published echocardiographic, computed tomography and cardiac MRI data (Stojanovska et al. 2011; Christiaens et al. 2009; Mahabadi et al. 2009; Hudsmith et al. 2007) when the exact method of analysis was applied. Main differences to the study were the method to evaluate the LA and LAA. Here, Hudsmith et al. (Hudsmith et al. 2007), who used cardiac MRI, included the LA appendage in the analysis, which therefore showed probably higher values. LA and LAA volumes and diameters are in line with those previously published (Stojanovska et al. 2011; Hudsmith et al. 2007). Guidelines from the American Society of Echocardiography have recommended LAV measurement to be used for LA size quantification in clinical practice (Calkins et al. 2012). As shown in previous studies (Stojanovska et al. 2011; Mahabadi et al. 2009; Hudsmith et al. 2007; Martinez 2009) LA diameter and volume are significantly influenced by BSA which indicates the need and appropriateness of indexing LA volume and diameter to BSA for reporting these parameters. Here, LAA volume and diameters were evaluated separately. Therefore, we evaluated absolute as well as indexed values in patients with paroxysmal and persistent AF as well as in patients with SEC and thrombus, showing significantly larger volumes and diameters in the LVES. However, the absolute as well as indexed values reported in this study are in line with those reported in previous published studies (Stojanovska et al. 2011; Christiaens et al. 2009; Mahabadi et al. 2009; Hudsmith et al. 2007; Martinez 2009). Intra/-interobserver variability’s were lower at LVES (39 ± 4% RR-interval; κ = 0.942) vs LVED (77 ± 5% RR-interval; κ = 0.891) in absolute values, it can be assumed due to larger LA/LAA volumes and diameters (p < 0.01) at LVES cardiac cycle.

### Prospective vs retrospective gating

In CCTA, prospective ECG-triggering needs overall less radiation dose than retrospective ECG-gating but serves less cardiac phases for evaluation (Menke et al. 2013).

Retrospective gating can be used with tube current modulation, where the full tube dose is applied e.g. at 30 - 70% of the RR-interval and is (prospectively ECG-based) reduced otherwise. Prospective gating is usable with temporal padding where more than a 180° half-scan is needed, e.g. to image the 70 - 80% RR-interval and to reconstruct the heart at 5% steps afterwards. The target of the exposure can therefore be set at 40% of the RR-interval (left atrium maximal volume) on a prospective trigger approach (Menke et al. 2013; Tsiflikas et al. 2010). Retrospective gating is preferable in patients with tachyarrhythmia, because CCTA images can be reconstructed at the required cardiac phase if the standard phase (LVED; 75% of RR-interval) was non-evaluatable (Menke et al. 2013). Prospective triggering is most appropriate in patients with regular normal heart rates and a maximum heart rate of about 75 beats/min. Discussing the general rules of radiation protection, at least in patients without tachyarrhythmia; CCTA could be performed with a prospectively triggered low-dose technique. Prospectively triggered CCTA should not be inferior to the more radiation-intense retrospectively gated method regarding the need for having reliable diagnostic information. Using a dual-source CT, all typical referral patients are mostly eligible for prospectively triggered CCTA due to the much faster image acquisition (e.g., 83 ms) while using 2 x-ray sources (Menke et al. 2013; Tsiflikas et al. 2010). However, this may not be suitable in patients with tachyarrhythmia, where even retrospectively gated CCTA with dual-source CT may be challenging (Sun et al. 2011).

### Limitations

In this study, we did not perform TEE or cardiac MRI measurements on the same subjects to compare the results with our LA/LAA diameters and volumes, but the results correlate with previously published LA/LAA diameters and values for MRI and TEE (Scherr et al. 2009; Stojanovska et al. 2011; Christiaens et al. 2009; Mahabadi et al. 2009; Hudsmith et al. 2007; Martinez 2009) obtained with comparable method of analysis. Previous studies (Stojanovska et al. 2011; Christiaens et al. 2009) have indicated that enlargement of the LA body is often not symmetric and that the antero-posterior LA linear dimension measurement may not be the most accurate and should not be used for risk stratification. Here, LA volume measurement is more reproducible and accurate for estimation of LA enlargement and may be predictive of cardiovascular outcome (Stojanovska et al. 2011). Physiologically, it can be assumed that LVES cardiac phase shows larger diameters in comparison to the LVED cardiac phase, therefore we performed measurements on 44 patients in this study as control group to proof this. TEE was considered the reference standard for thrombus and SEC detection. The absence or presence of LA thrombus was not confirmed by direct visual inspection of anatomic or surgical specimen. Despite that, TEE and CCTA were not performed the same day. However initial experience with a dual-source CT (DSCT) at our institution using the described contrast injection-protocol with a prospective trigger approach at LVES (39 ± 4% RR-interval) looks promising while significantly reducing radiation exposure to about <1-3 mSv.

## Conclusion

This is the first study to evaluate LA/LAA volumes and sizes in context of thrombus detection in patients prior to PVI at LVES and LVED cardiac cycle (39 ± 4% and 77 ± 5% RR-interval). Here the LVES (at 39 ± 4% RR-interval) reconstruction interval is recommended due to significantly larger LA/LAA diameters/volumes (p < 0.01) and lower intra/- interobserver variability’s (κ = 0.942 vs κ = 0.891). Quantitative analysis (threshold of < 0.5 when using LAA/AA ratio) of CCTA filling defects is superior to qualitative visual analysis in detecting or ruling out LA/LAA thrombus.
